# Maintenance of periarticular joint angles after varization double-level osteotomy for severe valgus deformity: a technical and radiographic mid-term case series

**DOI:** 10.1007/s00402-026-06539-2

**Published:** 2026-11-12

**Authors:** Moritz Herbst, Steffen Schröter, Atesch Ateschrang, Tina Histing, Marc-Daniel Ahrend

**Affiliations:** 1 Department of Traumatology and Reconstructive Surgery, BG Klinik Tübingen, Tübingen, Germany; 2 Department of Traumatology and Reconstructive Surgery, Diakonie Klinikum GmbH Jung-Stilling-Krankenhaus, Siegen, Germany; 3 Department of Orthopedics and Traumatology, KSA Kantonspital Aarau, Aarau, Switzerland

**Keywords:** Limb deformity, Joint orientation, Cartilage preservation, Gonarthrosis

## Abstract

**Purpose:**

To assess the restoration and mid-term course of mechanical alignment and periarticular femoral and tibial joint angles after varization double-level osteotomy (DLO) in patients with severe valgus malalignment.

**Methods:**

This retrospective case series included 13 knees treated with medial closing-wedge distal femoral and high tibial osteotomy between 2011 and 2015. Eight knees were available at a minimum follow-up of five years. Mechanical alignment and periarticular joint angles were assessed preoperatively, six weeks postoperatively, and at final follow-up. Individual radiographic trajectories, conversion to total knee arthroplasty, complications, and postoperative patient-reported outcomes were evaluated descriptively.

**Results:**

Seven patients with eight knees were assessed after a mean follow-up of 7.5 ± 1.2 years. Mean mTFA changed from 9.6 ± 2.6° valgus preoperatively to − 1.4 ± 1.5° six weeks postoperatively and 0.1 ± 4.7° at final follow-up. Mean mLDFA changed from 86.1 ± 2.2° to 90.5 ± 2.5° postoperatively and 90.2 ± 2.4° at final follow-up; mean mMPTA changed from 92.4 ± 1.6° to 86.4 ± 1.5° and 87.3 ± 2.1°, respectively. Individual trajectories were heterogeneous; one knee developed recurrent valgus alignment with a marked increase in JLCA. None of the eight knees underwent conversion to total knee arthroplasty. Three complications occurred, and implant removal was performed in all knees.

**Conclusion:**

Within the limitations of this small retrospective case series, varization DLO restored mechanical alignment and periarticular femoral and tibial joint angles in selected patients with severe valgus malalignment. Group-level periarticular bony angles remained close to the early postoperative values at mid-term follow-up, although individual radiographic trajectories were heterogeneous. Larger prospective comparative studies are required to determine clinical effectiveness, complication rates, survivorship, and valgus-specific indication criteria.

**Supplementary Information:**

The online version contains supplementary material available at https://doi.org/10.1007/s00402-026-06539-2.

## Introduction

Valgus malalignment of the knee is a clinically relevant condition that contributes to lateral compartment overload, meniscal pathology, and progressive chondral degeneration, accelerating the development and progression of lateral tibiofemoral osteoarthritis [8]. In younger and active patients, osteotomy to restore physiological joint angles remains an important joint-preserving treatment option, aiming to distribute load across the knee and delay the need for arthroplasty [22].

Recent morphological analyses have shown that valgus alignment is often bifocal, involving both the distal femur and proximal tibia [2]. If only one bone segment is corrected in such cases, substantial coronal correction may induce secondary deformities, including excessive joint-line obliquity (JLO), mediolateral subluxation, or non-physiological load distribution [33]. Maintaining a horizontal joint line is therefore critical for preventing abnormal cartilage loading and unfavorable postoperative biomechanics [33]. Double-level osteotomy (DLO) addresses this need by distributing the correction across both the femoral and tibial segments, thereby enabling precise mechanical alignment correction while preserving physiological joint-line orientation.

Historically, most evidence on DLO has emerged from studies on varus deformity, where high tibial osteotomy alone can result in pathological increases of the medial proximal tibial angle during large corrections. DLO effectively mitigates this risk and has demonstrated good accuracy and clinical outcomes in numerous clinical series [1, 3, 6, 20].

In contrast, clinical evidence on varization DLO for valgus knees has only recently emerged. Early reports suggest that DLO enables accurate correction of valgus deformities and results in meaningful improvements in patient-reported outcomes [15, 17, 21, 27]. However, the existing literature is limited by the small number of published cohorts, heterogeneity in surgical techniques, and predominantly short- to mid-term follow-up periods. As a result, the evidence base remains generally insufficient and considerably narrower than that for DLO in varus malalignment.

Given these limitations—and considering that valgus and varus deformities differ biomechanically, morphologically, and in their effect on joint-line convergence angle (JLCA) [17, 20, 21, 27]—there is a need for additional clinical data on the outcomes of varization DLO in valgus knees.

The primary aim of this retrospective case series was to assess the restoration and mid-term maintenance of mechanical alignment and periarticular femoral and tibial joint angles after varization double-level osteotomy in patients with severe valgus malalignment. Secondary aims were to descriptively evaluate individual radiographic changes, osteoarthritis progression, conversion to total knee arthroplasty, complications, and postoperative patient-reported outcomes at a minimum follow-up of five years. Given the exploratory nature and small sample size of the study, no confirmatory hypothesis was predefined.

## Materials and methods

### Study design and patient selection

This retrospective cohort study included patients who underwent a DLO between 2011 and 2015 at a level I trauma center. Participation was voluntary. A total of 12 patients (13 knees) underwent DLOs, consisting of a medial closing-wedge distal femoral osteotomy (DFO) and a medial closing-wedge high tibial osteotomy (HTO). The minimum follow-up duration was five years.

Of the 12 patients initially treated, five patients representing five knees were unavailable for final follow-up. Four patients could no longer be contacted, and one patient had died. Consequently, seven patients with eight treated knees were available for the final assessment after a mean follow-up of 7.5 ± 1.2 years (range, 5.7–9.4 years), corresponding to a patient follow-up rate of 58.3%. Available baseline characteristics of knees with and without final follow-up are presented descriptively in Table 1.

In the case where both limbs were treated, a minimum interval of one year was maintained between the two procedures to ensure complete recovery and independent rehabilitation.

The study was approved by the local ethics committee. Written informed consent was obtained from all participating patients prior to inclusion in the study.

**Table 1:** Patient characteristics

Characteristic	Final follow-up cohort	Knees without final follow-up
Number of patients	7	5
Number of knees	8	5
**Sex distribution by treated knee**		
Male	4 (50%)	1 (20%)
Female	4 (50%)	4 (80%)
**Affected side**		
Left	4 (50%)	1 (20%)
Right	4 (50%)	4 (80%)
Implant removal, n (%)	8 (100%)	Not available
Follow-up period [years]	7.5 ± 1.2 (5.7–9.4)	Not applicable
Age at surgery [years]	46.5 ± 13.7 (30.5–64.6)	41.4 ± 8.1 (31.0–50.0)
BMI [kg/m²]	31.1 ± 3.4 (27.4–37.1)	37.6 ± 6.6 (27.4–45.2)
Time to implant removal [years]	3.2 ± 2.9 (0.3–7.6)	Not available

Values are presented as mean ± standard deviation (range) or n (%)

### Indication and surgical technique

Varization DLO was considered in patients with symptomatic valgus malalignment and femoral and/or tibial deformity components. An mechanical medial proximal tibial angle (mMPTA) > 90° and an mechanical lateral distal femoral angle (mLDFA) < 85° were used as morphological indicators of tibial and femoral deformity, respectively, but were not applied as mandatory isolated thresholds. Preoperative planning included simulation of an isolated DFO or HTO according to the predominant deformity component. The target mechanical alignment was neutral to 2° of varus. DLO was selected when this target could not be achieved by a single-level correction without producing an additional non-physiological periarticular femoral or tibial angle. JLCA was evaluated as an intra-articular alignment parameter but was not used as a fixed bony planning threshold.

Detailed results of the individual single-level osteotomy simulations, including the limiting periarticular angle, simulated residual mechanical tibiofemoral angle (mTFA), and the rationale for selecting DLO, are provided in Supplementary Table S1.

One 51-year-old patient with a physically demanding occupation and continued sporting activity had advanced post-traumatic lateral compartment osteoarthritis after a lateral tibial plateau fracture. Arthroscopy confirmed grade 4 lateral cartilage damage, with only grade 1 changes in the medial and patellofemoral compartments. Simultaneous correction of the pronounced valgus deformity during primary knee arthroplasty was considered likely to result in collateral ligament instability and to require a constrained implant rather than an unconstrained surface-replacement prosthesis. Considering the patient’s age and functional demands, joint-preserving extra-articular realignment was selected to normalize the bony alignment and preserve the option of later unconstrained surface-replacement arthroplasty.

The surgical procedure is basically the same as described in detail by Kuwashima et al. [15]. Each DLO began with a diagnostic arthroscopy to evaluate cartilage status, confirm the indication, and exclude other diseases.

DLO was initiated with a minimally invasive biplanar medial closing-wedge distal femoral osteotomy, following the principles described by Lobenhoffer et al. 2017 [16] and van Heerwaarden et al. 2017 [31]. A 5–6 cm medial incision allowed subvastus access, with blunt preparation to the medial cortex. A radiolucent posterior retractor was positioned to protect neurovascular structures. Two to four K-wires were placed according to the preoperative plan to define the oblique transverse cut and the ascending cut, forming the typical biplanar configuration [16]. The transverse cut was oriented from proximal-medial to distal-lateral and confined to the posterior two-thirds of the femur to preserve an anterior cortical flange and minimize the risk of an anterior fracture [29]. The ascending cut measured approximately 5–6 cm. The medial wedge was removed, and the osteotomy was gently closed around the defined lateral hinge [29]. Fixation was achieved using an angular-stable plate (TomoFix^™^ plate; DePuy Synthes, Solothurn, Switzerland, medial distal femur (MDF) for distal femur) as described in previous closing-wedge DFO literature [9, 10].

The medial closing-wedge HTO was performed through a straight or slightly oblique incision, with careful preservation of the superficial medial collateral ligament (MCL), as the osteotomy lies distal to the deep-MCL insertion. A contoured retractor was used to limit soft-tissue detachment and enable safe access to the lateral hinge. As in the femur, the HTO was executed as a biplanar osteotomy, consisting of two precise oblique transverse cuts aimed toward the fibular head [13] followed by an ascending cut behind the patellar tendon. After removal of the wedge, the osteotomy was gently closed around the lateral hinge. Fixation was performed using a TomoFix^™^ medial high tibia (MHT) plate (DePuy Synthes, Solothurn, Switzerland) with locking screws [30]. Intraoperative alignment control was performed with fluoroscopy and an alignment rod to confirm the planned mechanical axis and joint-line orientation. Drains were placed, and wounds were closed in layers.

Postoperative management followed a standardized protocol with partial weight-bearing of 20 kg from postoperative day 1, progressing to full weight-bearing after approximately six weeks once early radiographic consolidation was observed. No orthoses were used; instead, active physiotherapy was applied.

### Outcome parameters

The primary outcome was the radiographic restoration and mid-term course of mechanical alignment and periarticular joint angles. Standardized radiological follow-up included anteroposterior and lateral knee radiographs and full-length weight-bearing anteroposterior radiographs obtained preoperatively, six weeks postoperatively, and at final follow-up. Radiographic measurements were performed using mediCAD^®^ digital planning software (Hectec, Landshut, Germany). The recorded parameters comprised the mTFA, mMPTA, mLDFA, JLCA, and JLO. Surgical accuracy was calculated according to established methods [7, 25]. Conversion to TKA, complications, and postoperative patient-reported outcomes were assessed descriptively as secondary outcomes.

Radiographic osteoarthritis severity in the medial, lateral, and patellofemoral compartments was graded according to the Kellgren–Lawrence classification [14] by two observers without blinding to patient information or assessment time point. For assessment of intrarater reliability, one observer repeated the grading after approximately six months. Linear-weighted Cohen’s kappa coefficients were calculated across all compartments and assessment time points. Because preoperative magnetic resonance imaging was not consistently available, intraoperative arthroscopic cartilage findings were additionally recorded for the medial and lateral compartments.

Clinical outcomes were evaluated through physical examination and validated functional scores, including the International Knee Documentation Committee (IKDC) score, the Knee Injury and Osteoarthritis Outcome Score (KOOS), the Oxford Knee Score (OKS), and the Lysholm score. Subjective health-related quality of life was assessed using SF-36 and compared with values for the normal population.

### Statistical analysis

Statistical analyses were performed using IBM^®^ SPSS^®^ Statistics version 31 for macOS. Given the small sample size, the radiographic analyses were considered exploratory and focused on descriptive statistics and individual radiographic trajectories. Continuous variables are reported as mean ± standard deviation and range. No adjustment was made for the non-independence of the two knees from one patient. Norm-based SF-36 scores were compared with the reference mean of 50 using one-sample t-tests. Intra- and interrater reliability of Kellgren–Lawrence grading were assessed using linear-weighted Cohen’s kappa coefficients with 95% confidence intervals. Statistical significance was set at *p* ≤ 0.05.

## Results

### Survival and complications

Patient characteristics and general data of the study population are shown in Table 1.

**Fig. 1:**
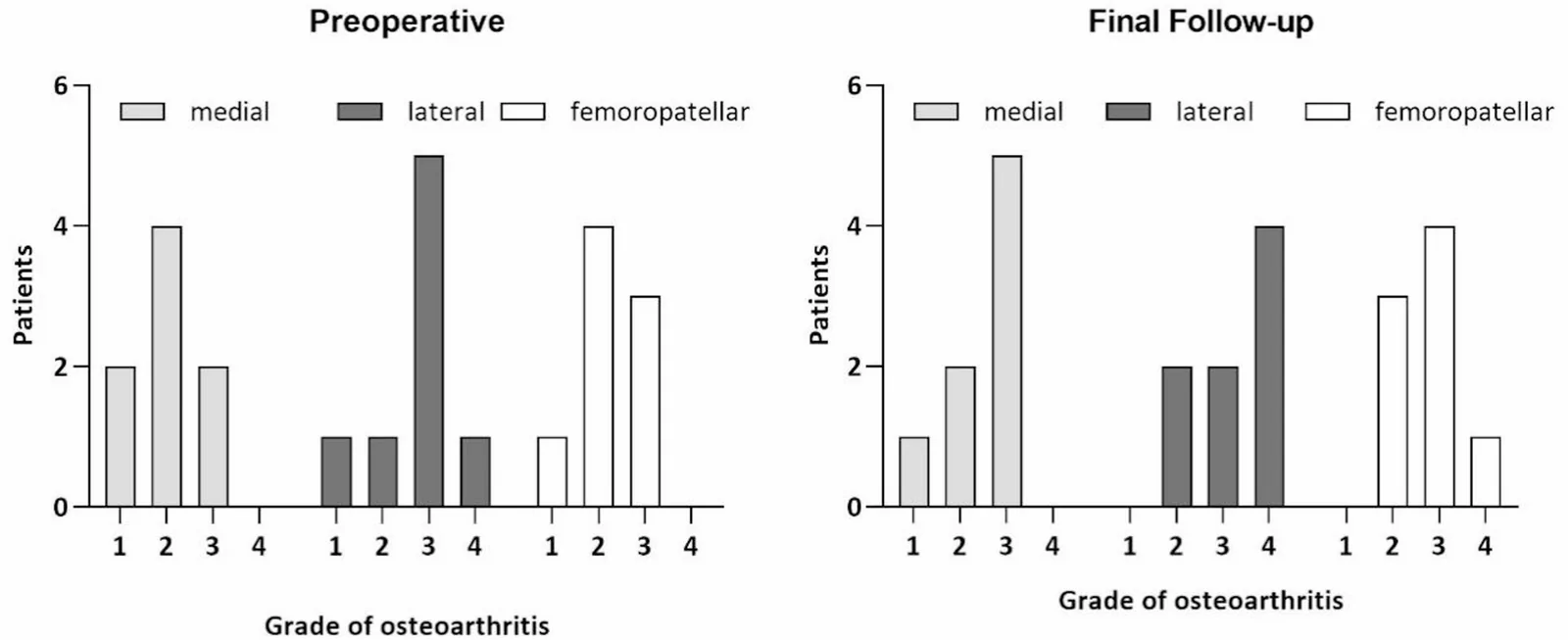
Distribution of osteoarthritis across knee joint compartments preoperatively and at final follow-up according to the Kellgren–Lawrence classification

Preoperative Kellgren–Lawrence grading showed lateral compartment osteoarthritis of grade I–II in two knees, grade III in five knees, and grade IV in one knee. Intraoperative arthroscopy demonstrated grade 3 or 4 cartilage damage in the lateral compartment of all eight knees, whereas medial cartilage damage ranged from grade 0 to 2. Radiographic changes during follow-up varied between compartments and individual knees and are shown descriptively in Fig. 1. Intrarater reliability of Kellgren–Lawrence grading was substantial, with a linear-weighted Cohen’s kappa of 0.737 (95% confidence interval, 0.602–0.871). Interrater reliability was moderate, with a linear-weighted Cohen’s kappa of 0.539 (95% confidence interval, 0.403–0.676).

At final follow-up, 7.5 ± 1.2 years postoperatively (range, 5.7–9.4 years), none of the eight knees had undergone conversion to TKA. Implant removal was performed in all eight knees after a mean of 3.2 ± 2.9 years (range, 0.3–7.6 years).

Three complications occurred among the eight knees available for final follow-up. One deep vein thrombosis was treated with temporary anticoagulation, one femoral hinge fracture was managed conservatively, and one post-traumatic wound infection required implant removal after three months. All three complications resolved without documented long-term sequelae.

### Radiological outcomes

Group-level and individual radiographic data are presented in Tables 2 and 3. Preoperatively, the knees showed a mean valgus mTFA of 9.6 ± 2.6° (range, 6.3–13.4°), accompanied by abnormal periarticular angles, with a mean mLDFA of 86.1 ± 2.2° (range, 82.9–89.5°) and a mean mMPTA of 92.4 ± 1.6° (range, 89.6–94.7°). Six weeks after DLO, the mean mTFA was − 1.4 ± 1.5° (range, − 4.1 to 0.3°), indicating neutral to slight varus alignment. The postoperative mean mLDFA was 90.5 ± 2.5° (range, 88.5–95.8°), and the mean mMPTA was 86.4 ± 1.5° (range, 84.2–88.1°).

**Table 2:** Radiographic parameters at preoperative assessment, preoperative planning, six weeks postoperatively, and final follow-up

Parameter	Preoperative	Planned	Postoperative	Final follow-up
mTFA [°]	9.6 ± 2.6 (6.3–13.4)	−0.7 ± 0.8 (− 2.1–0.5)	−1.4 ± 1.5 (− 4.1–0.3)	0.1 ± 4.7 (− 5.6–9.8)
mLDFA [°]	86.1 ± 2.2 (82.9–89.5)	91.0 ± 0.9 (90.0–92.5)	90.5 ± 2.5 (88.5–95.8)	90.2 ± 2.4 (88.1–95.5)
mMPTA [°]	92.4 ± 1.6 (89.6–94.7)	87.0 ± 1.5 (85.3–89.4)	86.4 ± 1.5 (84.2–88.1)	87.3 ± 2.1 (84.4–90.3)
JLO [°]	3.5 ± 3.2 (0.0–8.0)	–	2.6 ± 1.2 (1.0–4.0)	3.1 ± 1.7 (1.3–6.0)
JLCA [°]	3.3 ± 1.8 (0.6–5.6)	–	2.8 ± 1.8 (0.6–5.0)	3.8 ± 2.6 (0.6–9.1)

Values are presented as mean ± standard deviation (range). Negative mTFA values indicate varus alignment and positive values indicate valgus alignment

At group level, the postoperative values approximated the preoperative planning values for mTFA, mLDFA, and mMPTA. However, individual deviations were observed. The largest plan-to-postoperative deviation in mTFA occurred in knee 5, with a planned value of − 0.5° and a postoperative value of − 4.1°. The postoperative mLDFA of this knee was 95.8° compared with a planned value of 92.0°. These individual data preclude interpreting the group means as uniformly high correction accuracy. The planned femoral and tibial wedge heights were 5.4 ± 2.0 mm (range, 3.0–8.0 mm) and 6.3 ± 1.2 mm (range, 4.0–8.0 mm), respectively.

At final follow-up, mean mLDFA was 90.2 ± 2.4° (range, 88.1–95.5°), and mean mMPTA was 87.3 ± 2.1° (range, 84.4–90.3°), remaining close to the early postoperative values. Mean mTFA was 0.1 ± 4.7° (range, − 5.6 to 9.8°), but individual trajectories were heterogeneous. In knee 8, mTFA changed from − 0.3° postoperatively to 9.8° valgus at final follow-up, accompanied by an increase in JLCA from 3.0° to 9.1° and a change in mMPTA from 85.8° to 90.3°. At group level, mean JLO was 3.1 ± 1.7° (range, 1.3–6.0°), and mean JLCA was 3.8 ± 2.6° (range, 0.6–9.1°), although individual courses were heterogeneous.

**Table 3:** Individual radiographic parameters of the eight knees throughout the observation period

Parameter	Time point	1	2	3	4	5	6	7	8
mTFA [°]	Preoperative	6.3	8.4	12.7	10.3	13.4	7.5	7.2	11.0
	Planned	0.0	−0.6	0.5	−1.0	−0.5	−1.0	−2.1	−1.0
	Postoperative	−0.1	−1.9	0.3	−2.7	−4.1	−0.3	−1.8	−0.3
	Final follow-up	0.4	−5.6	2.8	−1.0	−4.1	0.4	−1.8	9.8
mLDFA [°]	Preoperative	87.1	87.1	82.9	86.7	83.3	86.8	89.5	85.3
	Planned	90.0	90.3	90.0	91.5	92.0	91.0	92.5	91.0
	Postoperative	88.9	88.9	88.5	92.3	95.8	89.5	90.8	89.1
	Final follow-up	88.7	88.3	88.1	91.6	95.5	89.4	90.7	89.6
mMPTA [°]	Preoperative	92.8	94.4	90.7	94.7	91.0	89.6	93.8	92.1
	Planned	89.4	88.5	85.6	88.1	86.0	85.3	87.5	85.8
	Postoperative	88.1	86.4	87.3	87.6	87.2	84.2	84.3	85.8
	Final follow-up	88.5	85.6	87.7	88.0	89.2	84.8	84.4	90.3
JLO [°]	Preoperative	1.9	1.3	7.5	1.5	8.0	1.8	0.0	6.2
	Postoperative	2.3	1.6	3.6	1.0	1.8	4.0	4.0	2.8
	Final follow-up	1.3	2.7	2.0	1.8	1.8	6.0	4.1	5.0
JLCA [°]	Preoperative	0.6	1.2	4.9	2.3	5.6	4.7	3.0	4.2
	Postoperative	0.7	0.6	1.6	2.0	4.5	5.0	4.7	3.0
	Final follow-up	0.6	2.8	3.2	2.6	2.2	5.0	4.6	9.1

Negative mTFA values indicate varus alignment and positive values indicate valgus alignment. Planned values were available for mTFA, mLDFA, and mMPTA only

### Clinical and functional results

**Fig. 2:**
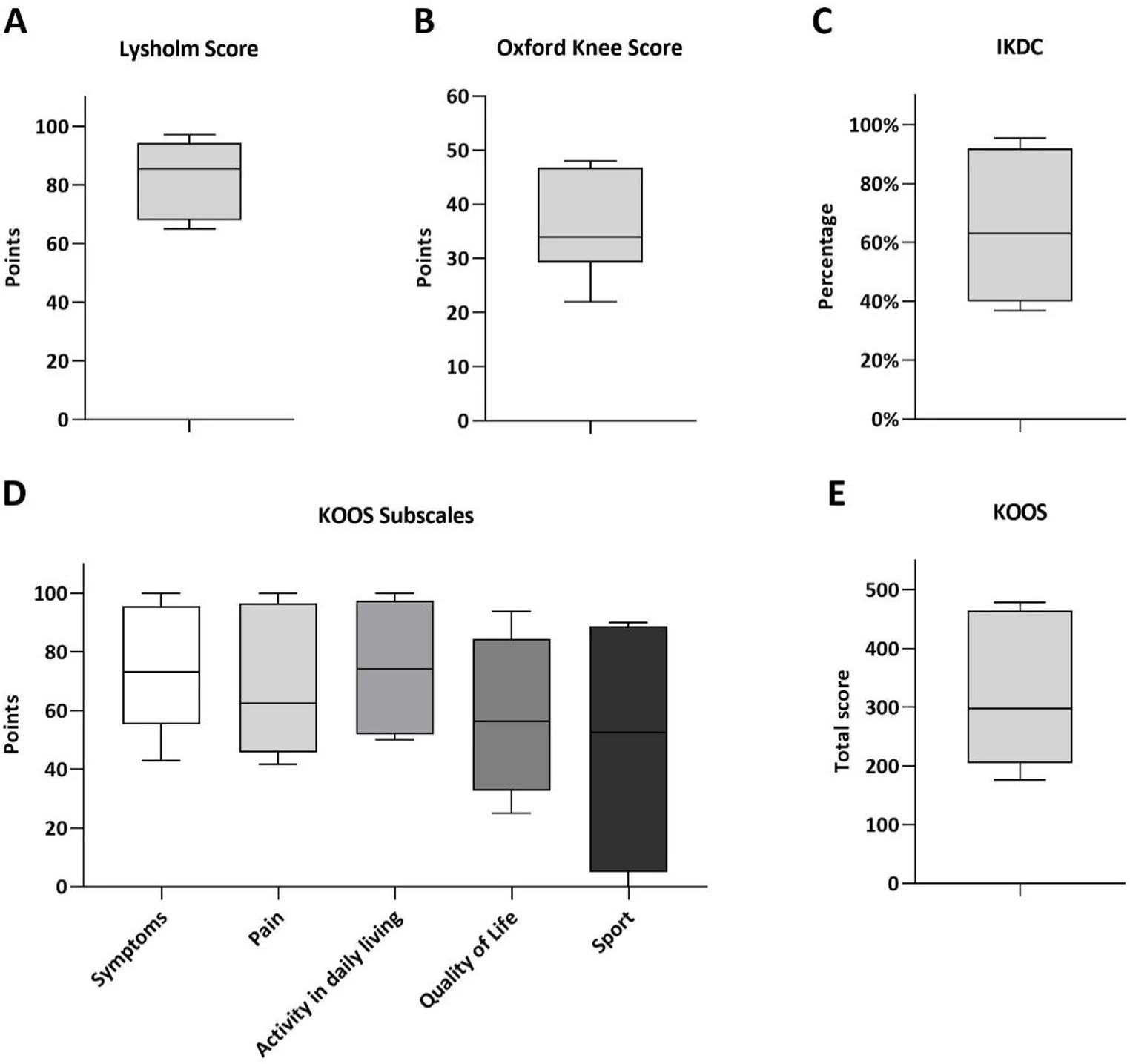
Boxplots. Results of clinical scores at the mid-term follow-up. **A** Lysholm Score results, **B** Oxford Knee Score results, **C** IKDC subjective results, **D** Results of individual subscales, **E** KOOS total score results. The upper and lower whiskers in the representation correspond to the 97.5th and 2.5th percentiles, respectively. Abbreviations: IKDC (International Knee Documentation Committee) subjective score, KOOS (Knee Injury and Osteoarthritis Outcome Scores)

The clinical and functional examination of the knee joints revealed no pathological findings regarding joint effusions, ligament stability, soft tissue conditions, or range of motion. The result for the Lysholm score was 82.5 ± 12.8 points (range, 65.0–97.0 points), for the OKS 36.2 ± 9.9 points (range, 22.0–48.0 points), and for the IKDC 65.2 ± 25.4% (range, 37.0–95.0%) (Fig. 2). The total KOOS score was 322.1 ± 129.3 points (range, 176.5–478.8 points), with the individual dimensions of pain, symptoms, daily activity, sport and leisure, and general quality of life achieving scores of 69.1 ± 24.6 points (range, 41.7–100.0 points), 73.2 ± 20.6 points (range, 42.9–100.0 points), 74.4 ± 23.6 points (range, 50.0–100.0 points), 47.5 ± 42.2 points (range, 0.0–90.0 points), and 57.8 ± 26.0 points (range, 25.0–93.8 points), respectively (Fig. 2).

The mean SF-36 physical component summary score was 38.2 ± 10.2 (range, 25.8–52.0), with lower norm-based scores for physical functioning and bodily pain than the reference mean of 50 (Table 4). The mean mental component summary score was 59.7 ± 11.2 (range, 36.5–70.4), while the mental-health-related subscales were generally close to or above the normative reference (Table 4).

**Table 4:** Norm-based SF-36 scores compared with the reference mean of 50

	SF-36	NP	Difference	*p* value
PCS Range	38.2 ± 10.2 (25.8–52.0)	50.0 ± 0.0	−11.8 ± 10.2*	0.014
PF Range	35.8 ± 17.6 (13.6–55.9)	50.0 ± 0.0	−14.2 ± 17.6	0.056
RP Range	51.6 ± 9.8 (28.1–56.9)	50.0 ± 0.0	1.6 ± 9.8	n.s.
BP Range	39.2 ± 6.4 (28.9–51.7)	50.0 ± 0.0	−10.7 ± 6.4*	0.002
GH Range	48.2 ± 12.3 (24.4–63.7)	50.0 ± 0.0	−1.8 ± 12.3	n.s.
MCS Range	59.7 ± 11.2 (36.5–70.4)	50.0 ± 0.0	9.7 ± 11.2*	0.044
V Range	51.4 ± 5.7 (43.3–60.0)	50.0 ± 0.0	1.4 ± 5.7	n.s.
SF ange	52.8 ± 9.4 (30.1–57.8)	50.0 ± 0.0	2.8 ± 9.4	n.s.
RE Range	49.9 ± 12.7 (18.6–55.3)	50.0 ± 0.0	−0.1 ± 12.7	n.s.
MH Range	60.4 ± 7.2 (47.1–65.6)	50.0 ± 0.0	10.4 ± 7.2*	0.005

Values are presented as mean ± standard deviation (range)

P values were calculated using two-sided one-sample t-tests against the norm-based reference mean of 50. *p ≤ 0.05

*PCS* physical health component summary score, *PF* physical functioning, *RP* physical role functioning, *BP* bodily pain, *GH* general health perception, *MCS* mental health component summary score, *V* vitality, *SF* social role functioning, *RE* emotional role functioning, *MH* mental health, *NP* normal population (adjusted for age and sex differences)

All patients reported being satisfied or even delighted with the treatment outcome (37.5% satisfied, 62.5% delighted). Patients’ expectations were met in 75.0% cases and exceeded in 25.0% cases. Ultimately, all patients stated that they would undergo the procedure again.

## Discussion

This exploratory case series shows that varization DLO can restore mechanical alignment and periarticular femoral and tibial joint angles in selected patients with severe valgus malalignment. At group level, the periarticular bony angles remained close to the early postoperative values at mid-term follow-up, although individual radiographic trajectories were heterogeneous. Postoperative patient-reported outcomes and satisfaction are reported descriptively because preoperative PROMs were unavailable.

Published clinical evidence on varization DLO remains limited to a few small, methodologically heterogeneous case series. These studies have described patient-specific guide-assisted or free-hand techniques, with short- to mid-term follow-up and varying radiographic and patient-reported outcomes [15, 17, 21, 27]. The present study adds mid-term individual and group-level radiographic data but does not resolve the limitations of the existing evidence base.

Previous small case series have reported generally close agreement between planned and achieved alignment after varization DLO using patient-specific guides or free-hand techniques [15, 21]. In the present cohort, postoperative group means approximated the planned values, but clinically relevant individual deviations occurred. Therefore, the data support the technical feasibility of free-hand DLO but do not establish uniformly high correction accuracy. The indication should remain individualized because validated valgus-specific thresholds for selecting DLO over SLO are currently lacking [15, 17, 21, 24].

Postoperatively, the mean mechanical limb axis was corrected to slight varus alignment, with an mTFA of − 1.4 ± 1.5° (range, − 4.1 to 0.3°), together with restoration of the periarticular knee angles, with an mMPTA of 86.4 ± 1.5° (range, 84.2–88.1°) and an mLDFA of 90.5 ± 2.5° (range, 88.5–95.8°). At final follow-up, the group means remained close to the postoperative values, although individual trajectories were heterogeneous. Evidence from isolated DFO suggests that excessive postoperative mLDFA may be associated with inferior clinical outcomes [26]. This observation supports considering DLO when simulated SLO would require disproportionate single-segment correction [26].

At group level, mean JLO and JLCA changed only modestly between the early postoperative and final follow-up assessments. However, individual trajectories were heterogeneous, and the small cohort does not permit conclusions regarding the biomechanical behavior of JLO or JLCA in valgus knees. In particular, one knee developed recurrent valgus alignment accompanied by a marked increase in JLCA, despite comparatively limited changes in the periarticular bony angles.

An increased JLO has been associated with greater cartilage shear stress and inferior clinical outcomes after correction of varus malalignment [20, 28]. Whether these findings can be transferred directly to valgus deformities remains uncertain. The present data support considering joint-line orientation during planning, but they neither establish a valgus-specific threshold nor demonstrate that maintenance of JLO improves clinical outcomes [17, 21].

Radiographic osteoarthritis was most pronounced in the lateral compartment before surgery. However, conclusions regarding compartment-specific progression are limited by the small cohort, the observer dependence of Kellgren–Lawrence grading, and the absence of systematic MRI follow-up [8, 21]. Although intrarater agreement was substantial, interrater agreement was only moderate. The arthroscopic findings confirmed advanced lateral cartilage damage with absent to moderate medial cartilage changes, but arthroscopy was available only at the time of surgery and therefore did not permit longitudinal assessment.

Clinical scores were available only at final follow-up and therefore describe postoperative status rather than treatment-related improvement. The observed scores indicate heterogeneous residual functional limitations, particularly for the IKDC score and the KOOS Sport and Quality of Life subscales. Nevertheless, all patients reported that their expectations were met or exceeded and stated that they would undergo the procedure again. Direct comparison with previous cohorts is limited by differences in patient selection, follow-up duration, surgical technique, and the absence of preoperative PROMs in the present study [15, 17, 21, 27].

The norm-based SF-36 scores provide a cross-sectional comparison with the age- and sex-adjusted reference population. Physical functioning and bodily pain remained below the normative reference, whereas the mental component score and several mental-health-related subscales were close to or above the reference mean. These findings describe health-related quality of life at final follow-up but cannot demonstrate a treatment-related change.

Short-term follow-up studies have reported no conversion to TKA following varization DLO [15, 21, 27]. Mabrouk et al. reported a conversion rate of 15.4% at mid-term follow-up after a mean of 4.9 ± 2.3 years [17]. In the present study, none of the eight knees available for final follow-up underwent conversion to TKA. Implant removal was performed in all eight knees after a mean of 3.2 ± 2.9 years (range, 0.3–7.6 years). Although implant removal was not classified as treatment failure, the 100% rate represents a relevant additional surgical burden and should be discussed during preoperative patient counseling [3, 17].

Although all complications resolved without documented long-term sequelae, the small cohort does not allow a reliable estimate of complication rates or procedural safety. Similar complications have been reported after osteotomies around the knee [12, 15, 17, 18, 21, 27, 32].

### Limitations

This study has several limitations. First, the retrospective case series design without a control group limits the level of evidence and does not allow direct comparison with alternative treatment strategies such as SLO or joint replacement procedures. Clinical outcome scores were available only at final follow-up, precluding longitudinal comparison across different time points. However, radiological data were available for all assessment time points and were analyzed accordingly.

Second, the small final cohort limits generalizability and precludes robust conclusions regarding long-term outcomes. Five of the original 12 patients were unavailable for final assessment, resulting in a patient follow-up rate of 58.3% and a substantial risk of selection bias. Patients returning for follow-up may therefore not be representative of the initial cohort. Available baseline characteristics were compared descriptively: patients without final follow-up were younger on average, with a mean age of 41.4 ± 8.1 years (range, 31.0–50.0 years) versus 46.5 ± 13.7 years (range, 30.5–64.6 years), but had a higher mean BMI of 37.6 ± 6.6 kg/m² (range, 27.4–45.2 kg/m²) versus 31.1 ± 3.4 kg/m² (range, 27.4–37.1 kg/m²). These opposing differences do not allow an inference regarding the direction of potential selection bias. No inferential comparison was performed because of the small sample size and limited available baseline data.

In addition, Kellgren–Lawrence assessments were not blinded to patient information or assessment time point, which may have introduced observer bias. The moderate interrater agreement further limits the precision of conclusions regarding compartment-specific osteoarthritis progression.

## Conclusion

Within the limitations of this small retrospective case series, varization DLO restored mechanical alignment and periarticular femoral and tibial joint angles in selected patients with severe valgus malalignment. At mid-term follow-up, the group-level periarticular bony angles remained close to the early postoperative values, although individual radiographic trajectories were heterogeneous. None of the eight knees available for final follow-up had undergone conversion to TKA; this descriptive observation does not provide a robust estimate of survivorship.

These findings support the technical feasibility of distributing correction across the distal femur and proximal tibia in selected cases where simulated single-level osteotomy would require disproportionate correction of one bone segment. Larger prospective comparative studies are needed to determine clinical effectiveness, complication rates, survivorship, and valgus-specific indication criteria.

## Supplementary Information

Below is the link to the electronic supplementary material.

Supplementary Material 1

## Data Availability

The datasets generated during and/or analyzed during the current study are available from the corresponding author on reasonable request.
