# Supplementary material for: Maintenance of periarticular joint angles after varization double-level osteotomy for severe valgus deformity: a technical and radiographic mid-term case series
Source: Arch Orthop Trauma Surg. 2026 Nov 12;146(1):403. doi: 10.1007/s00402-026-06539-2 (PMC13759443; doi:10.1007/s00402-026-06539-2)
Supplement: Supplementary file 1 [file 402_2026_6539_MOESM1_ESM.docx]

**Supplementary Table S1: Individual simulation of single-level osteotomy and rationale for selecting double-level osteotomy**

| **Knee** | **Simulated single-level procedure** | **Limiting postoperative angle** | **Simulated residual mTFA** | **Reason for DLO selection** |
| --- | --- | --- | --- | --- |
| 1 | Isolated DFO | mLDFA 90.0° | 3.4° valgus | Residual valgus with combined femoral and tibial deformity |
| 2 | Isolated HTO | mMPTA 85.0°; preoperative mLDFA 87.1° | 0.7° valgus | Target alignment could not be achieved without further tibial correction in a combined deformity |
| 3 | Isolated DFO | mLDFA 90.0° | 5.6° valgus | Residual valgus with combined femoral and tibial deformity |
| 4 | Isolated DFO | mLDFA 92.0° | 5.6° valgus | Relevant residual valgus despite femoral correction |
| 5 | Isolated DFO | mLDFA 92.0° | 4.6° valgus | Residual valgus; joint-preserving realignment selected in a 51-year-old active patient with post-traumatic lateral grade 4 cartilage damage |
| 6 | Isolated DFO | mLDFA 91.0° | 3.4° valgus | Residual valgus after limited femoral correction |
| 7 | Isolated HTO | mMPTA 85.0° | 0.2° valgus | Planned target was 2° varus; further isolated tibial correction would have required a more pronounced tibial angle |
| 8 | Isolated DFO | mLDFA 91.0° | 5.3° valgus | Relevant residual valgus despite femoral correction |
